# Safety and 1‐Year Outcomes After Transplanting Hearts From SARS‐CoV‐2 Positive Donors: Insights From an International Analysis

**DOI:** 10.1002/iid3.70252

**Published:** 2025-09-05

**Authors:** Sabina P. W. Guenther, Josephine Wadewitz, Brian J. Wayda, Henrik Fox, Rayan Cheaban, Yasuhiro Shudo, William Hiesinger, Angelika Costard‐Jäckle, Michiel Morshuis, Y. Joseph Woo, Jeffrey J. Teuteberg, René Schramm, Axel Rahmel, Jan F. Gummert, Kiran K. Khush

**Affiliations:** ^1^ Clinic for Thoracic and Cardiovascular Surgery, Heart and Diabetes Center North Rhine‐Westphalia Ruhr‐University Bochum Bad Oeynhausen Germany; ^2^ German Organ Procurement Organisation (DSO) Germany; ^3^ Division of Cardiovascular Medicine Stanford University School of Medicine Stanford California USA; ^4^ Department of Cardiothoracic Surgery Stanford University School of Medicine Stanford California USA

**Keywords:** COVID‐19 donors, donor characteristics, heart transplantation, SARS‐CoV‐2 donors, transplant outcomes

## Abstract

**Background:**

Uncertainties persist regarding the utilization of hearts from SARS‐CoV‐2‐positive donors for heart transplant (HT). This international study analyzed such HTs within the United States (US) and Germany, focusing on 1‐year outcomes and granular safety data.

**Methods:**

Data was obtained from the United Network for Organ Sharing (UNOS) registry (03/2021–08/2022) and collaborating with the German Organ Procurement Organisation (DSO; 03/2022–02/2023). HTs from currently and recently (up to 21 days in UNOS and 90 days in DSO) SARS‐CoV‐2‐positive donors were included.

**Results:**

In the US, 274 HTs from SARS‐CoV‐2 donors were analyzed (50.7% SARS‐CoV‐2‐positive until organ recovery). Compared to 3952 HTs from SARS‐CoV‐2‐negative donors, acute rejection was less frequent (10.6% vs. 17.1%, *p* = 0.006). One‐year graft and recipient survival (*p* = 0.327) and rehospitalization rates (*p* = 0.592) did not differ. In Germany, 30 HTs utilized SARS‐CoV‐2‐positive hearts. Follow‐up was obtained for 23 (76.7%). 43.5% of the donors were positive until recovery. Two recipients (8.7%) tested positive for SARS‐CoV‐2 21 and 65 days post‐transplant, both unlikely donor‐derived. 8.7% had severe PGD, 8.7% acute cellular rejection ≥ 2R. One‐year survival was 91.3%. None experienced myocarditis or thromboembolism.

**Conclusion:**

Using selected SARS‐CoV‐2‐positive hearts for transplant appears safe with no differences in 1‐year survival, no evidence of viral transmission or SARS‐CoV‐2‐related adverse cardiovascular events.

## Graphical abstract

An international analysis within the US and Germany demonstrates that using selected hearts from donors with active or recent SARS‐CoV‐2‐infection for transplant is safe, with no differences in 1‐year survival, no evidence of viral transmission or SARS‐CoV‐2‐related adverse cardiovascular events.

**Abbreviations**
BMI: body mass index
CPR: cardiopulmonary resuscitation
CT: computed tomography
Ct: cycle threshold
CVA: cerebrovascular accident
CXR: chest X‐ray
c‐pos: currently positive
DSO: German Organ Procurement Organisation
ECMO: extracorporeal membrane oxygenation
EF: ejection fraction
ET: Eurotransplant
FU: follow‐up
HT: heart transplant
HU: high urgency
ICH: intracranial hemorrhage
ICM: ischemic cardiomyopathy
IQR: interquartile range
LV: left ventricle
MCS: mechanical circulatory support
NAT: nucleic acid amplification testing
NICM: nonischemic cardiomyopathy
NOS: not otherwise specified
n.a.: not available
PGD: primary graft dysfunction
RV: right ventricle
r‐pos: recently positive
T: transplantable
UNOS: United Network for Organ Sharing
US: United States
VAD: ventricular assist device

## 1 Introduction

Internationally, no evidence‐based consensus exists on the utilization of donor hearts from SARS‐CoV‐2 positive donors for transplant due to a paucity of outcome and safety data. Therefore, as we now face an endemic situation, the evaluation of such donor hearts remains associated with uncertainties and challenges.

Initial case series and studies with limited cohort sizes or follow‐up times suggested acceptable short‐term outcomes after utilizing hearts from SARS‐CoV‐2 positive donors [1, 2, 3, 4, 5]. However, a preliminary analysis of the United Network for Organ Sharing (UNOS) registry reported increased 6‐month and 1‐year mortality in recipients of hearts from actively COVID‐19 positive donors compared to recipients of COVID‐negative donor hearts, which prompted caution when considering hearts from SARS‐CoV‐2 positive donors for transplant [6]. Nonetheless, granular data on these transplants and follow‐up beyond the short‐term remain limited.

The virus has tropism for the respiratory tract [7]. Yet, virus particles have been detected in blood and several non‐respiratory tissues, including the myocardium [6, 7, 8]. Further, adverse cardiovascular events including dysrhythmias, myocarditis, heart failure, and thromboembolism have been reported during and months after SARS‐CoV‐2 infection [6, 9, 10, 11]. The mechanisms underlying the association between SARS‐CoV‐2 infection and the development of cardiovascular diseases are not well understood, and the impact of immunosuppression of heart transplant (HT) recipients on these pathophysiological pathways remains unclear [7, 9].

Here, we present an international study that analyzes outcomes after transplanting hearts from currently or recently SARS‐CoV‐2 positive donors within the United States (US) and German centers within Eurotransplant (ET) and focuses on 1‐year clinical outcomes, including granular data on safety.

## 2 Materials and Methods

The UNOS registry was queried for HT utilizing SARS‐CoV‐2 positive donors, and transplant volumes were compared to ET. As outcome data are limited in the ET registry, we focused on HT in Germany, wherein HT using SARS‐CoV‐2 positive donors were analyzed and prospectively followed in collaboration with the German Organ Procurement Organisation (DSO). For these HT, the donor SARS‐CoV‐2 status was systematically characterized, including repeat nucleic acid amplification testing (NAT) results, cycle threshold (Ct) values, symptoms, and chest imaging findings. Next, transplanting centers were queried on recipient‐level SARS‐CoV‐2 related factors, such as vaccination status and peri‐operative management. Outcome data, including adverse cardiovascular events, were queried at hospital discharge, 6 months, and 1 year after HT.

Inclusion criteria for both cohorts were adult HT (defined as donor age ≥ 18 years in the German cohort and recipient age ≥ 18 years in the US cohort) with available information on donor SARS‐CoV‐2 status by antigen test and/or NAT from upper or lower respiratory sources. Multiorgan transplants were excluded.

Data on SARS‐CoV‐2 testing results were available up to 21 days before organ recovery within UNOS, and up to 90 days before organ recovery within DSO. To assess safety and outcomes of both, HT from currently and recently SARS‐CoV‐2 positive donors, we stratified donors into three categories of positivity: (1) Positive at organ recovery (currently positive, c‐pos group), (2) recently positive, within less than 7 days of donation, but negative at the time of recovery (r‐pos (< 7 d) group), and (3) recently positive, from 7 up to 21 days (UNOS) or 90 days (DSO) of donation, respectively, but negative at the time of recovery (r‐pos (≥ 7 d) group).

Guidance and policies on the allocation of hearts from SARS‐CoV‐2 positive donors varied internationally, resulting in differing study periods for the cohorts. In the US, a significant number of HT utilizing SARS‐CoV‐2 positive donors was only recorded as of March 2021. Therefore, all HTs as of March 01, 2021, were included in this study. Data were available for transplants through August 31, 2022. Follow‐up data were available through August 31, 2023, enabling 1‐year outcome analyses for the entire cohort.

On January 25, 2022, the first donor with a positive test for SARS‐CoV‐2 was reported from Belgium to the ET office in Leiden for organ allocation. The first SARS‐CoV‐2 positive donor from Germany was reported to the ET office for allocation on February 25, 2022. Therefore, HTs from March 1, 2022 until February 28, 2023 were included. Prospective outcome analyses were performed until 1 year after transplant.

The primary endpoint for both cohorts was a composite of mortality and graft failure. Secondary endpoints for both cohorts were duration of hospitalization after HT, acute rejection, and rehospitalizations. For UNOS, differences in outcomes between HT utilizing SARS‐CoV‐2 positive versus negative donor hearts were analyzed. Additional endpoints in DSO included virus transmission, primary graft dysfunction (PGD), arrhythmias, myocarditis, and thromboembolic events.

### 2.1 Statistical Analysis

Categorial variables are presented as percentages and absolute numbers, and continuous variables as medians with interquartile ranges. Data were available for all HT unless otherwise indicated. The Kaplan–Meier estimator was used to assess time to the primary endpoint, and statistical differences were determined using the log‐rank test. For group comparisons, the *t*‐test and the *χ* ^2^ test were used as appropriate. The study was approved by the Institutional Review Boards of the Heart and Diabetes Center North Rhine‐Westphalia, Bad Oeynhausen, Germany and Stanford University School of Medicine, USA.

## 3 Results

**Figure 1:**
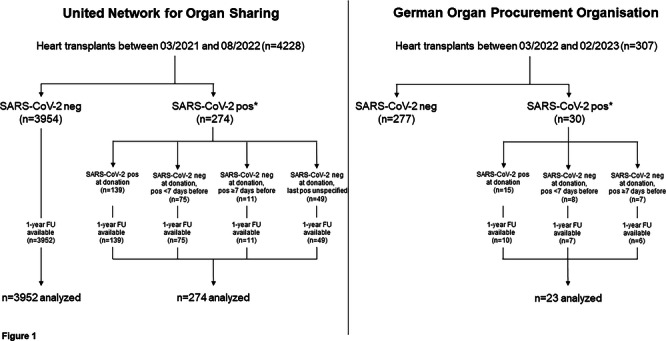
Study flowchart. *SARS‐CoV‐2 positivity was defined as current positivity at the time of organ donation or recent positivity shortly before organ donation. For recent positivity, data on SARS‐CoV‐2 testing results were available up to 21 days before organ recovery within the United Network for Organ Sharing registry, and up to 90 days before organ recovery within the German Organ Procurement Organisation data set. FU, follow‐up.

The study cohorts are presented in Figure 1. Altogether, 297 HTs utilizing hearts from currently or recently SARS‐CoV‐2 positive donors were analyzed, with a cumulative follow‐up of 408.1 patient years.

### 3.1 United Network for Organ Sharing

#### 3.1.1 Study Cohort

From March 2021 to August 2022, 21,441 potential donors were recorded in the UNOS database, and 4228 hearts were utilized for transplant. One‐year follow‐up data were available for 4226 HTs, and these were further analyzed. A total of 274 hearts (6.5%) were from currently or recently SARS‐CoV‐2 positive donors.

#### 3.1.2 Donor Characteristics

The median age of all currently or recently SARS‐CoV‐2 positive donors (*n* = 274) was 30.0 (23.0–37.0) years, and 20.1% were female. The cohort was further stratified into: (1) donors with a positive result on the latest SARS‐CoV‐2 test before recovery (currently positive donors, c‐pos group; *n* = 139, median age 30.0 (22.0–36.0) years, 20.9% female), (2) donors who were negative at the time of recovery, but recently positive less than 7 days of donation (r‐pos (< 7 d) group; *n* = 75, median age 31.0 (25.0–37.0) years, 18.7% female), and (3) donors who were negative at the time of recovery, but recently positive from 7 to 21 days of donation (r‐pos (≥ 7 d) group; *n* = 11, median age 28.0 (20.0–39.0) years, 18.2% female). Additionally, within the UNOS cohort, 49 donors were negative at the time of recovery, but positive within the 21 days before, with unknown timing of the last positive test (r‐pos (unspecified) group; median age 30.0 (22.0–39.0) years, 20.4% female).

Further details are given in Table 1. A relevant number of donors in all subgroups had at least one cardiovascular risk factor, such as diabetes, hypertension, smoking history, or obesity, with obesity being the most common. Of all SARS‐CoV‐2 positive donors, 9.4% had undergone cardiopulmonary resuscitation efforts, with a range of 7.7%–30.0% within the subgroups.

**Table 1:** Donor and recipient baseline characteristics stratified by SARS‐CoV‐2 status.

	Currently and recently SARS‐CoV‐2‐positive donors UNOS					Currently and recently SARS‐CoV‐2‐positive donors DSO			
	All (*n* = 274)	Currently pos (50.7%, *n *=139)	Currently neg, pos < 7 d ago (27.4%, *n *= 75)	Currently neg, pos ≥ 7 d ago (4.0%, *n *= 11)	Currently neg, last pos unspecified (17.9%, *n *= 49)	All (*n *= 23)	Currently pos (43.5%, *n *= 10)	Currently neg, pos < 7 d ago (30.4%, *n *= 7)	Currently neg, pos ≥ 7 d ago (26.1%, *n *= 6)
**Donor**									
Age (years)	30.0 (23.0–37.0)	30.0 (22.0–36.0)	31.0 (25.0–37.0)	28.0 (20.0–39.0)	30.0 (22.0–39.0)	44.0 (25.0–54.0)	46.5 (23.0–53.0)	47.0 (32.0–57.0)	35.0 (24.0–45.0)
BMI (kg/m^2^)	26.0 (23.0–31.0)	26.0 (23.0–31.0)	26.0 (23.0–32.0)	28.0 (22.0–30.0)	26.0 (23.0–32.0)	26.0 (23.0–33.0)	25.0 (21.5–29.8)	28.0 (25.0–33.0)	24.0 (21.5–27.8)
Female sex	20.1 (55)	20.9 (29)	18.7 (14)	18.2 (2)	20.4 (10)	65.2 (15)	70.0 (7)	42.9 (3)	83.3 (5)
Sex mismatch^b^	18.6 (51)	16.6 (23)	22.7 (17)	9.1 (1)	20.4 (10)	34.8 (8)	30.0 (3)	42.9 (3)	33.3 (2)
Cause of death									
CVA/ICH	7.3 (20)	5.0 (7)	13.3 (10)	18.2 (2)	2.0 (1)	43.5 (10)	50.0 (5)	42.9 (3)	33.3 (2)
Head trauma	42.0 (115)	43.9 (61)	41.3 (31)	27.3 (3)	40.8 (20)	8.7 (2)	0 (0)	0 (0)	33.3 (2)
Anoxia	46.7 (128)	47.5 (66)	38.7 (29)	54.5 (6)	55.1 (27)	39.1 (9)	40.0 (4)	57.1 (4)	16.7 (1)
Cerebral edema (NOS)	0 (0)	0 (0)	0 (0)	0 (0)	0 (0)	8.7 (2)	10.0 (1)	0 (0)	16.7 (1)
Other	4.0 (11)	3.6 (5)	6.7 (5)	0 (0)	2.0 (1)	0 (0)	0 (0)	0 (0)	0 (0)
S/p CPR	9.4 (24/255^a^)	7.7 (10/130^a^)	9.7 (7/72^a^)	30.0 (3/10^a^)	9.3 (4/43^a^)	47.8 (11)	60.0 (6)	71.4 (5)	0 (0)
Cardiac risk factors									
Diabetes	5.2 (14/267^a^)	3.0 (4/134^a^)	5.4 (4/74^a^)	10.0 (1/10^a^)	10.2 (5)	8.7 (2)	20.0 (2)	0 (0)	0 (0)
Hypertension	12.0 (32/267^a^)	9.7 (13/134^a^)	16.2 (12/74^a^)	0 (0/10^a^)	14.3 (7)	21.7 (5)	10.0 (1)	42.9 (3)	16.7 (1)
Smoking^c^	13.7 (36/262^a^)	11.4 (15/132^a^)	19.2 (14/73^a^)	11.1 (1/9^a^)	12.5 (6/48^a^)	43.5 (10)	50.0 (5)	28.6 (2)	50.0 (3)
Obesity	31.8 (87)	30.2 (42)	36.0 (27)	36.4 (4)	28.6 (14)	30.4 (7)	30.0 (3)	42.9 (3)	16.7 (1)
LVEF < 50%	1.1 (3)	0 (0)	2.7 (2)	0 (0)	2.0 (1)	4.5 (1/22^a^)	0 (0)	16.7 (1/6^a^)	0 (0)
RV function abnormal	n.a.	n.a.	n.a.	n.a.	n.a.	0 (0/22^a^)	0 (0)	0 (0/6^a^)	0 (0)
SARS‐CoV‐2 specifics									
Latest NAT Ct value	—	n.a	—	—	—	—	37.5 (32.7–40.2)	—	—
Symptomatic	n.a.	n.a.	n.a.	n.a.	n.a.	31.6 (6/19^a^)	25.0 (2/8^a^)	0 (0/6^a^)	80.0 (4/5^a^)
Abnormal CXR/CT findings	n.a.	n.a	n.a.	n.a.	n.a.	50.0 (10/20^a^)	50.0 (4/8^a^)	50.0 (3/6^a^)	50.0 (3)
**Recipient**									
Age at transplant (years)	56.0 (44.0–63.0)	55.0 (43.0–63.0)	57.0 (48.0–65.0)	59.0 (36.0–66.0)	56.0 (47.0–65.0)	43.0 (18.0–54.0)	52.0 (22.0–55.0)	38.0 (20.0–61.0)	23.5 (14.0–45.5)
Female sex	21.9 (60)	23.0 (32)	22.7 (17)	27.3 (3)	16.3 (8)	34.8 (8)	40.0 (4)	14.3 (1)	50.0 (3)
Blood type									
A	36.5 (100)	37.4 (52)	34.7 (26)	36.4 (4)	36.7 (18)	30.4 (7)	40.0 (4)	42.9 (3)	0 (0)
B	11.7 (32)	10.1 (14)	17.3 (13)	18.2 (2)	6.1 (3)	0 (0)	0 (0)	0 (0)	0 (0)
AB	2.9 (8)	2.9 (4)	1.3 (1)	9.1 (1)	4.1 (2)	0 (0)	0 (0)	0 (0)	0 (0)
0	48.9 (134)	49.6 (69)	46.7 (35)	36.4 (4)	53.1 (26)	69.6 (16)	60.0 (6)	57.1 (4)	100.0 (6)
Etiology of heart failure									
ICM	27.7 (76)	30.2 (42)	17.3 (13)	54.6 (6)	30.6 (15)	21.7 (5)	20.0 (2)	28.6 (2)	16.7 (1)
NICM	67.2 (184)	64.8 (90)	76.0 (57)	45.5 (5)	65.3 (32)	65.2 (15)	70.0 (7)	71.4 (5)	50.0 (3)
Congenital disease	5.1 (14)	5.0 (7)	6.7 (5)	0 (0)	4.1 (2)	13.0 (3)	10.0 (1)	0 (0)	33.3 (2)
Re‐transplant (graft failure)	0 (0)	0 (0)	0 (0)	0 (0)	0 (0)	0 (0)	0 (0)	0 (0)	0 (0)
Prior cardiac surgery	37.7 (100/265^a^)	38.1 (51/134^a^)	37.0 (27/73^a^)	36.4 (4)	38.3 (18/47^a^)	56.5 (13)	60.0 (6)	42.9 (3)	66.7 (4)
S/p durable VAD	28.1 (77)	29.5 (41)	22.7 (17)	27.3 (3)	32.7 (16)	39.1 (9)	50.0 (5)	14.3 (1)	50.0 (3)
Inotrope‐dependent	39.4 (108)	40.3 (56)	42.7 (32)	27.3 (3)	34.7 (17)	30.4 (7)	20.0 (2)	57.1 (4)	16.7 (1)
Temporary MCS‐dependent	37.6 (103)	39.6 (55)	40.0 (30)	18.2 (2)	32.7 (16)	17.4 (4)	10.0 (1)	42.9 (3)	0 (0)
Waitlist status before HT									
1	8.4 (23)	7.2 (10)	9.3 (7)	9.1 (1)	10.2 (5)	—	—	—	—
2	46.0 (126)	46.0 (64)	46.7 (35)	45.5 (5)	44.9 (22)	—	—	—	—
3	15.7 (43)	15.1 (21)	16.0 (12)	18.2 (2)	16.3 (8)	—	—	—	—
4+	29.9 (82)	31.7 (44)	28.0 (21)	27.3 (3)	28.6 (14)	—	—	—	—
T	—	—	—	—	—	22.7 (5/22^a^)	40.0 (4)	16.7 (1/6^a^)	0 (0)
HU	—	—	—	—	—	77.3 (17/22^a^)	60.0 (6)	83.3 (5/6^a^)	100.0 (6)

*Note:* Values are provided as median (IQR) or % (*n*).

Abbreviations: BMI, body mass index; CPR, cardiopulmonary resuscitation; Ct, cycle threshold; CT, computed tomography; CVA, cerebrovascular accident; CXR, chest X‐ray; HT, heart transplantation; HU, high urgent; ICH, intracranial hemorrhage (any); ICM, ischemic cardiomyopathy; LVEF, left ventricular ejection fraction; n.a., not available; NAT, nucleic acid amplification testing; NICM, non‐ischemic cardiomyopathy; NOS, not otherwise specified; RV, right ventricle; T, transplantable; temporary MCS, temporary mechanical circulatory support (e.g., intra‐aortic balloon pump, Impella (Abiomed, Danvers, USA), extracorporeal membrane oxygenation); VAD, ventricular assist device.

^a^ Data availability as indicated.

^b^ Defined as a female donor to male recipient constellation.

^c^ Defined as “cigarette use > 20 pack years ever” in UNOS and as “anytime smoking” in DSO.

In the same study period, a total of 3952 hearts from SARS‐CoV‐2 negative donors were transplanted. When compared to all currently or recently SARS‐CoV‐2 positive donors, negative donors were slightly older, more often female, and differed in causes of death (as further detailed in Supporting Information S1: Table S1).

#### 3.1.3 Recipient Characteristics

The median age of all patients receiving a heart from a currently or recently SARS‐CoV‐2 positive donor was 56.0 (44.0–63.0) years, and 21.9% were female. Recipients of c‐pos donor hearts were 55.0 (43.0–63.0) years old (23.0% female), recipients of r‐pos (< 7 d) donor hearts 57.0 (48.0–65.0) years (22.7% female), recipients of r‐pos (≥ 7 d) donor hearts 59.0 (36.0–66.0) years (27.3% female), and recipients of r‐pos (unspecified) donor hearts 56.0 (47.0–65.0) years (16.3% female), respectively.

Details on the causes of heart failure are given in Table 1, with nonischemic cardiomyopathy (NICM) being the predominant etiology. Of all the recipients, most were listed as status 2 before HT (46.0%), followed by status 4–6 (29.9%), status 3 (15.7%), and status 1 (8.4%). More than a third had prior cardiac surgical procedures (37.7%, *n* = 100/265), and 28.1% were on durable ventricular assist device (VAD) support. Further, 39.4% were inotrope‐dependent (range for the subgroups 27.3%–42.7%), and 37.6% were supported with temporary mechanical circulatory support (MCS; range for the subgroups 18.2%–40.0%).

When comparing recipients of hearts from currently or recently SARS‐CoV‐2 positive donors to recipients of hearts from SARS‐CoV‐2 negative donors, blood type 0 was more common among recipients of hearts from positive donors, and these recipients were more frequently on durable VAD support as bridge to transplant (as further detailed in Supporting Information S1: Table S1).

#### 3.1.4 Safety and Outcomes

Acute rejection (any acute rejection requiring treatment) during index hospitalization was less frequent in recipients of hearts from currently or recently SARS‐CoV‐2 positive donors than in recipients of hearts from negative donors (10.6% vs. 17.1%, *p*= 0.006, Supporting Information S1: Table S1).

After discharge, there were no differences in rehospitalization rates for any cause (*p* = 0.592), for rejection (*p* = 0.871), and for infection (*p* = 0.091).

**Figure 2:**
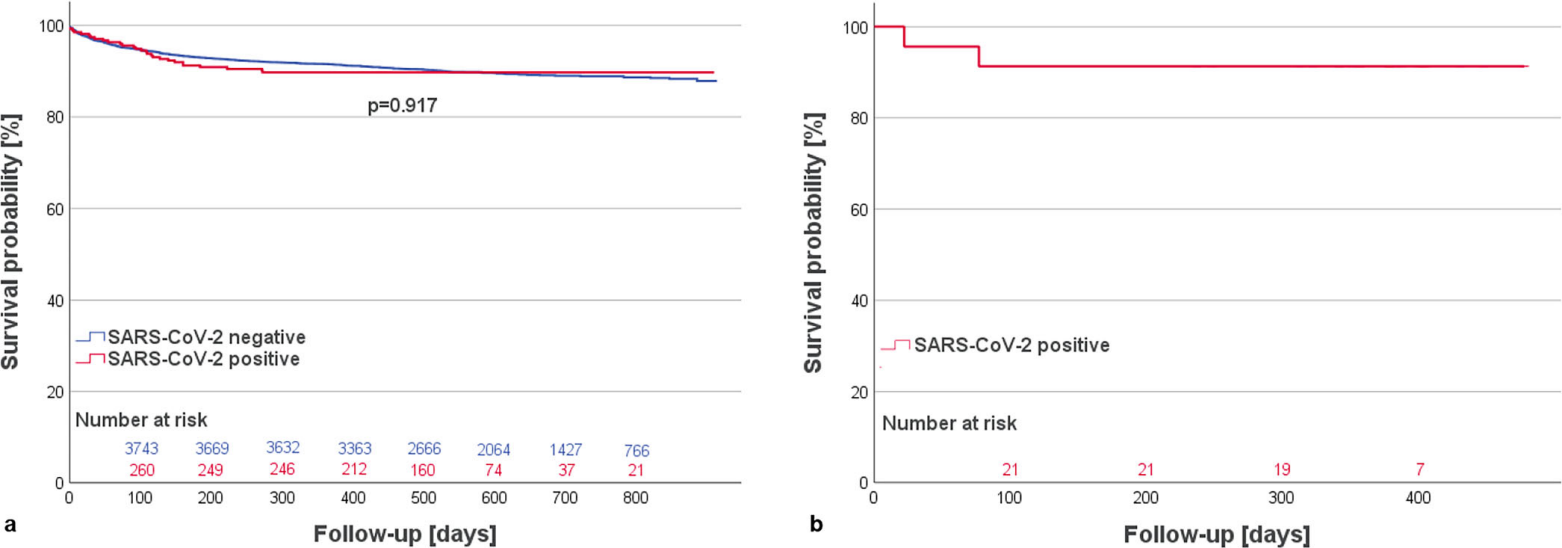
Composite endpoint of graft and recipient survival. (a) US cohort: Comparison between recipients of hearts from currently or recently SARS‐CoV‐2 positive versus negative donors. (b) German cohort: Recipients of hearts from currently or recently SARS‐CoV‐2 positive donors.

The composite primary endpoint of death and graft failure is depicted in Figure 2. The total median follow‐up is 543.5 (406.5–618.3) days for recipients of SARS‐CoV‐2 positive donor hearts, and 616.0 (456.0–765.0) days for recipients of negative donor hearts. We did not observe any differences in death and graft failure at 1 year between recipients of c‐pos and r‐pos donor hearts versus recipients of donor hearts from SARS‐CoV‐2 negative donors (10.2% vs. 8.5%, *p* = 0.327). The same holds true when comparing the c‐pos group versus negative donors only (10.1% vs. 8.5%, *p* = 0.082).

### 3.2 German Organ Procurement Organisation in Eurotransplant

#### 3.2.1 Study Cohort

From March 2022 to February 2023, a total of 402 potential donor hearts were reported by DSO to the ET office, and 307 (76.4%) of them were transplanted. Of these, 30 (9.8%) were from currently or recently SARS‐CoV‐2 positive donors. Follow‐up was obtained for 23 (76.7%), and these HTs were further analyzed.

#### 3.2.2 Donor Characteristics

he median donor age was 44.0 (25.0–54.0) years, and 65.2% were female. The cohort was similarly stratified: (1) donors with a positive result of the latest SARS‐CoV‐2 test before recovery (currently positive donors, c‐pos group; *n* = 10, median age 46.5 (23.0–53.0) years, 70.0% female), (2) donors that were negative at the time of recovery, but recently positive less than 7 days of donation (r‐pos (< 7 d) group; *n* = 7, median age 47.0 (32.0–57.0) years, 42.9% female), and (3) donors that were negative at the time of recovery, but recently positive from 7 up to 90 days of donation (r‐pos (≥ 7 d) group; *n *= 6, median age 35.0 (24.0–45.0) years, 83.3% female).

Cardiovascular risk factors were common in all groups (Table 1). Diagnostic workup showed normal biventricular function in all but one case (95.5%, data availability *n *= 22/23), where the left ventricular (LV) function was mildly reduced. Coronary angiograms were performed in 43.5% (*n* = 10), with abnormal results in two (luminal irregularities/stenoses < 25% and luminal irregularities/stenoses < 25% plus 26%–50% distal left anterior descending stenosis, respectively).

Donors last tested positive 2.0 (1.0–11.0) days before organ recovery (range 0–90 days). For c‐pos donors (*n* = 10), the latest Ct value was 37.5 (32.7–40.2, range 30.8–41.5). In three of the c‐pos donors, the Ct value was slightly decreasing, with, however, Ct values still greater 30 before recovery in all. In another three donors, no judgement on Ct dynamics was possible as tests had been performed on the same day (one donor), or only one test had been obtained (two donors). Another three donors were positive in the latest test pre‐recovery after previous tests were negative. One donor showed an increasing Ct value. Information on COVID‐related symptoms was available for 82.6% (*n* = 19/23): 31.6% of these were symptomatic at the time of recovery or had been symptomatic at the time of recent SARS‐CoV‐2 infection (25.0% of c‐pos, 0% of r‐pos (< 7 d), and 80.0% of r‐pos (≥ 7 d) donors, respectively). Thoracic imaging (chest x‐ray or computed tomography) was available for 87.0% (*n* = 20/23), with potentially pneumonia‐related or residual pathological findings in 50.0% of these (50.0% of c‐pos, 50.0% of r‐pos (< 7 d), and 50.0% of r‐pos (≥ 7 d) donors, respectively). However, it was not possible to clearly differentiate between COVID‐related pathological findings and abnormalities secondary to other causes, for example, aspiration.

#### 3.2.3 Recipient Characteristics

The median age of all recipients was 43.0 (18.0–54.0) years, and 34.8% were female. Recipients of c‐pos donor hearts were 52.0 (22.0–55.0) years old (40.0% female), recipients of r‐pos (< 7 d) donor hearts 38.0 (20.0–61.0) years (14.3% female), and recipients of r‐pos (≥ 7 d) donor hearts 23.5 (14.0–45.5) years (50.0% female), respectively.

Similar to US recipients, NICM was the most frequent cause of end‐stage heart failure (Table 1). About half of the patients had been operated on before (56.5% of all recipients, 60.0% of c‐pos recipients, 42.9% of r‐pos (< 7 d) recipients, and 66.7% of r‐pos (≥ 7 d) recipients, respectively), and 39.1% of all recipients were on durable VAD support (50.0% of c‐pos recipients, 14.3% of r‐pos (< 7 d) recipients, and 50.0% of r‐pos (≥ 7 d) recipients, respectively). The majority (77.3%, data availability *n* = 22/23) were listed as status high urgency (HU) before HT, whereas 19.0% were INTERMACS level 1, 4.8% INTERMACS level 2, and 76.2% INTERMACS level 3 or lower (data availability *n* = 21/23). Altogether, 30.4% were inotrope and 17.4% temporary MCS‐dependent.

Most recipients (95.5%, data availability *n* = 22/23) were fully vaccinated against COVID‐19 (as defined by local authorities at the time of HT).

#### 3.2.4 Safety and Outcomes

None of the recipients received peri‐operative pharmacological prophylaxis against SARS‐CoV‐2 infection. No alterations to the centers' standard immunosuppression regimens were made, except in one case (4.5%, data availability *n* = 22/23).

In 91.3%, postoperative surveillance testing for SARS‐CoV‐2 was performed at the time of transplant, that is, the likelihood of detecting even inapparent infections was high. During the index hospitalization, two patients (8.7%) tested positive for SARS‐CoV‐2 on days 21 (r‐pos (< 7 d) recipient) and 65 (c‐pos recipient) after HT (Table 2). Both were asymptomatic and in both, donor‐derived transmission was deemed unlikely by the transplanting center.

**Table 2:** Outcomes stratified by SARS‐CoV‐2 status.

	Currently and recently SARS‐CoV‐2‐positive donors UNOS					Currently and recently SARS‐CoV‐2‐positive donors DSO			
	All (*n *= 274)	Currently pos (50.7%, *n *= 139)	Currently neg, pos < 7 d ago (27.4%, *n *= 75)	Currently neg, pos ≥ 7 d ago (4.0%, *n* = 11)	Currently neg, last pos unspecified (17.9%, *n *= 49)	All (*n *= 23)	Currently pos (43.5%, *n *= 10)	Currently neg, pos < 7 d ago (30.4%, *n *= 7)	Currently neg, pos ≥ 7 d ago (26.1%, *n *= 6)
**Index hospitalization**									
SARS‐CoV‐2 infection	n.a.	n.a.	n.a.	n.a.	n.a.	8.7 (2)	10.0 (1)	14.3 (1)	0 (0)
Graft dysfunction	n.a.	n.a.	n.a.	n.a.	n.a.	13.0 (3)	20.0 (2)	14.3 (1)	0 (0)
Acute rejection^b^	10.6 (29)	10.1 (14)	9.3 (7)	18.2 (2)	12.2 (6)	8.7 (2)	0 (0)	14.3 (1)	16.7 (1)
Hospital stay (days)	17.0 (12.0–27.0)	17.0 (12.0–28.0)	16.0 (11.0– 24.0)	26.0 (12.0–52.0)	18.0 (15.0–27.0)	38.0 (24.0–57.0)	53.0 (29.5–76.3)	36.0 (19.0–54.0)	29.5 (22.5–43.5)
**1‐year follow‐up**									
Any rehospitalization	44.3 (86/194^a^)	46.2 (42/91^a^)	39.1 (25/64^a^)	44.4 (4/9^a^)	50.0 (15/30^a^)	23.8 (5/21^a^)	0 (0/9^a^)	50.0 (3/6^a^)	33.3 (2)
For rejection	5.2 (10/194^a^)	7.7 (7/91^a^)	3.1 (2/64^a^)	0 (0/9^a^)	3.3 (1/30^a^)	0 (0/21^a^)	0 (0/9^a^)	0 (0/6^a^)	0 (0)
For infection	10.8 (21/194^a^)	12.1 (11/91^a^)	4.7 (3/64^a^)	22.2 (2/9^a^)	16.7 (5/30^a^)	9.5 (2/21^a^)	0 (0/9^a^)	33.3 (2/6^a^)	0 (0)
Composite death/graft failure	10.2 (28)	10.1 (14)	13.3 (10)	9.1 (1)	6.1 (3)	8.7 (2)	10.0 (1)	14.3 (1)	0 (0)

*Note:* Values are provided as median (IQR) or % (*n*).

Abbreviation: n.a., not available.

^a^ Data availability as indicated.

^b^ Defined as acute cellular rejection ≥ ISHLT 2 R and/or requiring treatment, or acute humoral rejection requiring treatment.

Altogether, three patients (13.0%) showed postoperative graft dysfunction, with severe primary graft dysfunction occurring on the day of HT and requirement of extracorporeal membrane oxygenation (ECMO) support in two (8.7%; *n* = 1 c‐pos, *n* = 1 r‐pos (< 7 d), respectively). At discharge, both these patients had normal LV function, while the right ventricular (RV) function was mildly reduced in one. The third patient (c‐pos) experiencing postoperative graft dysfunction showed unspecified RV dysfunction on postoperative Day 2 and required tricuspid valve repair 4 days later. The patient was discharged with normal LV and mildly reduced RV function.

Acute cellular rejections ≥ ISHLT 2R were observed in 8.7% (*n* = 2). No cases of antibody‐mediated rejections were observed. Atrial fibrillation was recorded in 8.7% (*n* = 2), and other arrhythmias in 13.0% (*n* = 3; third‐degree atrioventricular block, sinus node dysfunction, chronotropic incompetence/intermittent junctional rhythm). No cases of thrombosis, embolism, or myocarditis were observed.

The in‐hospital mortality was 8.7% (*n* = 2). Causes of death were intracranial hemorrhage 22 days after HT with an otherwise uneventful course, and acute respiratory distress syndrome with requirement of venovenous ECMO support and eventually sepsis and multiorgan failure 77 days after HT. In the latter, no evidence of SARS‐CoV‐2 infection was reported. All remaining patients (*n* = 21) had a normal LV function at discharge, with a mildly reduced RV function in 19.0% (*n* = 4/21), which includes the two patients with postoperative graft dysfunction as detailed above.

No patients expired after discharge, and no cases of graft failure occurred, resulting in 91.3% 1‐year recipient and graft survival. The total median follow‐up was 378.0 (323.0–403.0) days.

One recipient (4.8%) experienced an episode of mildly reduced allograft function (LV ejection fraction 46%) 2 months after HT with normal biventricular function at 1‐year follow‐up. Further, no episodes of acute cellular rejection ≥ ISHLT 2R or antibody‐mediated rejection, no cases of thrombosis, embolism, myocarditis, or arrhythmias were observed. Five recipients (23.8%) required rehospitalization for seven episodes: COVID‐19 pneumonia 4 months after HT, HSV pneumonia, vertigo, rule out of pulmonary embolism and fluid overload, norovirus infection, spontaneous intramuscular hematoma due to vitamin K antagonist intake, and pericardial effusion after endomyocardial biopsy, respectively. The case of COVID‐19 pneumonia was not reported to be considered donor‐derived by the transplanting center.

At 1 year, all patients had normal LV function, with mildly reduced RV function in 15.0% (*n* = 3, data availability *n* = 20/21).

## 4 Discussion

Our study aimed to assess the safety and 1‐year outcomes after transplantation of hearts from currently or recently SARS‐CoV‐2 positive donors. This is the first study to combine large‐scale registry and SARS‐CoV‐2 specific granular data, and the first study to present international data. Our key findings are: (1) 1‐year recipient and graft survival did not differ between recipients of hearts from SARS‐CoV‐2 positive donors and those receiving hearts from SARS‐CoV‐2 negative donors; (2) there was no evidence of donor‐derived viral transmission; (3) there was no increase in incidence of acute rejection; and (4) there was no evidence of adverse cardiovascular events such as recipient myocarditis or thromboembolism.

In the early phase of the pandemic, SARS‐CoV‐2 donor hearts were not considered for transplant. As time elapsed, recommendations evolved, and organ procurement organizations as well as HT centers became less restrictive. This was the result of a better understanding of the virus and its pathophysiology, increasing vaccination rates, candidate exposure due to previous illness, availability of potential therapeutics, and the publication of preliminary reports on safety and robust outcomes after using SARS‐CoV‐2 positive donor hearts [1, 2, 8, 12, 13, 14, 15]. However, utilization is still frequently limited to presumably very low or low risk SARS‐CoV‐2 positive donors (i.e., donors that subsequently tested negative before organ recovery, donors with high Ct values, or asymptomatic donors) [1, 2, 4, 16, 17]. Not utilizing or underutilizing SARS‐CoV‐2‐positive donor hearts bears the risk of underutilizing the available total donor pool [2, 6, 14]. Thus, as we have reached an endemic situation, evidence regarding the safety of utilizing organs from currently or recently infected donors has become increasingly relevant.

The main areas of concern are potential donor‐derived viral transmission, compromised graft function or survival, and the risk of adverse cardiovascular events.

Donor‐derived virus transmission poses a particular risk in the setting of post‐HT immunosuppression. Moreover, a poor immune response to SARS‐CoV‐2 messenger RNA vaccines has been observed in a relevant percentage of transplant recipients, potentially rendering this cohort more vulnerable to the virus [18, 19]. Importantly, we did not observe any cases of suspected donor‐derived viral transmission, and no such case has been reported in HT recipients to date. So far, insufficient data are available on candidate vaccination policies, and on peri‐operative antiviral prophylaxis against SARS‐CoV‐2 infection, resulting in a lack of international standards. We queried DSO centers on their practices regarding both, and the vast majority of recipients were fully vaccinated at the time of HT, while no specific prophylaxis was used in any of these cases.

Our UNOS cohort analysis of 274 recipients of SARS‐CoV‐2 positive donor hearts did not show differences in graft and recipient survival at 1 year in comparison to 3952 recipients of negative hearts. Notably, this is the first analysis to have a complete 1‐year follow‐up. The same results were obtained when comparing c‐pos recipients to negative recipients only. Although no direct control group was available for the German cohort, national quality indicators are frequently published. One‐year survival between 2012 and 2019 ranged from 72.8% to 84.6%, and the 1‐year survival within our cohort (91.3%) is well above that [20]. These findings support the trend reported by Wolfe et al. who analyzed a similar UNOS data set but with a limited follow‐up time [21], and are in contrast to the results by Madan et al. who reported increased 6‐month and 1‐year mortality for recipients of HTs from active COVID‐19 donors (NAT positive within 2 days of procurement) compared to non‐COVID donors [6]. The cohort analyzed by Madan et al., however, was smaller, and the follow‐up time was even shorter, with a median follow‐up for HTs from COVID‐19 donors of 5.7 (1.5–6.5) months [6]. Consequently, the study was only able to examine 1‐year outcomes for a limited number of HTs using COVID‐19 donors (*n* < 50). Our results, in contrast, are the first to provide complete 1‐year follow‐up data on a large cohort (*n* = 274) of HTs that used currently or recently SARS‐CoV‐2 positive donors. This allowed for a robust assessment of 1‐year outcomes, showing no differences in graft and recipient survival.

Further, rehospitalization rates in our UNOS analyses were similar between recipients of SARS‐CoV‐2‐positive and negative donor hearts, which is indicative of similar morbidity and post‐HT adverse event rates in the mid‐ to long‐term. This is underscored by the absence of major complications, including severe graft dysfunction after hospital discharge, for up to 1 year after HT in the German cohort.

Lastly, in this analysis, no evidence of potentially SARS‐CoV‐2‐related adverse cardiovascular events, such as recipient myocarditis or thromboembolism, was seen in the German cohort. Although we observed some cases of arrhythmias during the index hospitalization after HT, no further arrhythmias were observed thereafter for up to 1 year.

Interestingly, acute rejection during index hospitalization was less frequent in recipients of hearts from currently or recently SARS‐CoV‐2 positive donors than in recipients of hearts from negative donors. A true biological correlation—that is, protection against rejection due to SARS‐CoV‐2 infection—seems very unlikely. The most likely explanation appears to be a random, spurious association, given that the longer‐term outcomes in the sense of rehospitalization rates for rejection in the first year were not significantly different. However, we cannot rule out other causes, such as unobserved differences in recipient characteristics (e.g., level of sensitization, or other rejection risk factors) between groups, or differences between centers. For example, some centers that used hearts from SARS‐CoV‐2 positive donors may have a higher experience or a lower risk‐aversion and be less likely to treat and label borderline cases as rejection.

Lastly, discussions have arisen regarding how to proceed with the routine testing of donors for SARS‐CoV‐2, and future consensus and recommendations will be necessary. As this study shows, an asymptomatic or minimally symptomatic SARS‐CoV‐2 infection does not contraindicate the use of a donor heart. Current evidence suggests the same is true for other non‐lung organs. Therefore, we believe that routine SARS‐CoV‐2 testing can be omitted for asymptomatic non‐lung donors.

### 4.1 Limitations

This study has several strengths. It is the first to combine international data from the US and Germany, to present a complete 1‐year follow‐up for the largest COVID‐positive donor cohort study in HT to date, and to add safety‐related data at the donor, recipient, and outcome‐level. However, the study also has several limitations. The study periods, inclusion of recently positive (≥ 7 d) donors, and follow‐up parameters varied between the UNOS and DSO cohorts due to data availability. “Adult HT” was defined as donor age ≥ 18 years in the German cohort and as recipient age ≥ 18 years in the US cohort. The cohort sizes are still limited and long‐term results beyond 1 year are not yet available. Due to the limited cohort size, only descriptive analyses were used for the German cohort. Additionally, follow‐up within this cohort reached a substantial 76.7%, but unfortunately, it did not reach 100%.

It is possible that donor inclusion favored “lower‐risk” positive cases, such as donors with high or increasing Ct‐values. In part, this likely stems from initial recommendations and regulations. However, it limits applicability of the findings to all SARS‐CoV‐2 positive donors. Future studies should address stratification based on donor viral load, clinical symptoms, or recipient immunologic risk. Further, one aspect that remains poorly answered is the safety of utilizing grafts from donors who died due to COVID‐19. None of the donors in the DSO cohort had died of COVID‐19, and none suffered from severe COVID‐19‐associated acute respiratory distress syndrome, while this information was unavailable for donors in the UNOS cohort. The most severe acute infections might confer additional risk that is not reflected in this study, and consideration of these hearts for transplantation must be performed with great caution.

## 5 Conclusions

To summarize, this study combines international large‐scale registry and granular data, and demonstrates the safety of using selected SARS‐CoV‐2‐positive donor hearts for transplantation. One‐year recipient and graft survival was similar between those receiving hearts from SARS‐CoV‐2 positive and negative donors. There was no evidence of viral transmission, greater risk of acute rejection, recipient myocarditis, or thromboembolism.

## Author Contributions

**Josephine Wadewitz:** conceptualization, data curation, formal analysis, investigation, methodology, project administration, validation, writing – original draft, writing – review and editing. **Brian J. Wayda:** conceptualization, data curation, formal analysis, investigation, methodology, validation, writing – original draft, writing – review and editing. **Henrik Fox:** conceptualization, formal analysis, methodology, writing – review and editing. **Rayan Cheaban:** conceptualization, investigation, methodology, validation, writing – review and editing. **Yasuhiro Shudo:** conceptualization, formal analysis, investigation, methodology, validation, writing – review and editing. **William Hiesinger:** conceptualization, formal analysis, methodology, validation, writing – review and editing. **Angelika Costard‐Jäckle:** conceptualization, formal analysis, investigation, methodology, validation, writing – review and editing. **Michiel Morshuis:** conceptualization, investigation, methodology, validation, writing – review and editing. **Y. Joseph Woo:** conceptualization, investigation, methodology, validation, writing – review and editing. **Jeffrey J. Teuteberg:** conceptualization, formal analysis, investigation, methodology, validation, writing – original draft. **René Schramm:** conceptualization, formal analysis, investigation, methodology, validation, writing – review and editing. **Axel Rahmel:** conceptualization, data curation, formal analysis, investigation, methodology, project administration, resources, supervision, validation, writing – original draft, writing – review and editing. **Jan F. Gummert:** conceptualization, formal analysis, investigation, methodology, resources, supervision, validation, writing – review and editing. **Kiran K. Khush:** conceptualization, data curation, formal analysis, investigation, methodology, project administration, resources, supervision, validation, writing – original draft.

## Conflicts of Interest

The authors declare no conflicts of interest.

## Supporting information


**Supplement 1:** Comparison between currently and recently SARS‐CoV‐2 positive and negative donors in UNOS.

## Data Availability

Because of the sensitive nature of these data, the data sets cannot be provided directly by us to other researchers for purposes of reproducing the results or replicating the procedure.
