# Supplementary material for: Safety and 1‐Year Outcomes After Transplanting Hearts From SARS‐CoV‐2 Positive Donors: Insights From an International Analysis
Source: Immun Inflamm Dis. 2025 Sep 5;13(9):e70252. doi: 10.1002/iid3.70252 (PMC12412413; doi:10.1002/iid3.70252)
Supplement: Supplementary file 1 — Supplement 1: Comparison between currently and recently SARS‐CoV‐2 positive and negative donors in UNOS. [file IID3-13-e70252-s001.docx]

|  | **Currently and recently**  **SARS-CoV-2 positive donors UNOS** | **SARS-CoV-2**  **negative donors**  **UNOS** |  |
| --- | --- | --- | --- |
|  | **All**  **(n=274)** | **All**  **(n=3952)** | **p** |
| **Donor** |  |  |  |
| Age [years] | 30.0 (23.0-37.0) | 32.0 (25.0-40.0) | **<0.001** |
| BMI [kg/m^2^] | 26.0 (23.0-31.0) | 27.0 (24.0-31.0) | 0.423 |
| Female sex  Sex mismatch* | 20.1 (55)  18.6 (51) | 27.5 (1087)  19.7 (780) | **0.008**  0.654 |
| Cause of death  CVA/ICH  Head trauma  Anoxia  Cerebral edema (NOS)  Other | 7.3 (20)  42.0 (115)  46.7 (128)  0 (0)  4.0 (11) | 12.4 (490)  39.1 (1547)  46.4 (1833)  0 (0)  2.1 (82) | **0.012**  0.355  0.915  -  **0.034** |
| S/p CPR | 9.4 (24/255^#^) | 7.8 (287/3663^#^) | 0.366 |
| Cardiac risk factors  Diabetes  Hypertension  Smoking**  Obesity | 5.2 (14/267^#^)  12.0 (32/267^#^)  13.7 (36/262^#^)  31.8 (87) | 4.0 (157/3892^#^)  16.5 (642/3886^#^)  12.8 (488/3820^#^)  31.8 (1255) | 0.335  0.051  0.649  0.996 |
| LVEF <50% | 1.1 (3) | 1.5 (58) | 0.618 |
|  | | | |
| **Recipient** |  |  |  |
| Age at transplant [years] | 56.0 (44.0-63.0) | 57.0 (46.0-64.0) | 0.205 |
| Female sex | 21.9 (60) | 27.1 (1071) | 0.061 |
| Blood type  A  B  AB  0 | 36.5 (100)  11.7 (32)  2.9 (8)  48.9 (134) | 38.8 (1535)  15.2 (599)  4.8 (189)  41.2 (1629) | 0.440  0.119  0.158  **0.013** |
| Etiology of heart failure  ICM  NICM  Congenital disease  Re-transplant (graft failure) | 27.7 (76)  67.2 (184)  5.1 (14)  0 (0) | 27.9 (1102)  67.4 (2664)  4.6 (181)  0.1 (5) | 0.948  0.940  0.685  - |
| Prior cardiac surgery  S/p durable VAD | 37.7 (100/265^#^)  28.1 (77) | 34.8 (1331)  22.9 (904) | 0.338  **0.047** |
| Inotrope-dependent | 39.4 (108) | 39.4 (1557) | 0.997 |
| Temporary MCS-dependent | 37.6 (103) | 41.0 (1619) | 0.271 |
| Waitlist status prior to HT  1  2  3  4+ | 8.4 (23)  46.0 (126)  15.7 (43)  29.9 (82) | 10.7 (421)  51.0 (2014)  13.7 (542)  24.7 (975) | 0.239  0.109  0.357  0.052 |
|  | | | |
| **Outcomes** |  |  |  |
| **Index hospitalization** |  |  |  |
| Acute rejection*** | 10.6 (29) | 17.1 (674) | **0.006** |
| Hospital stay [days] | 17.0 (12.0-27.0) | 17.0 (12.0-26.0) | 0.636 |
| **1-year follow-up** |  |  |  |
| Any rehospitalization  For rejection  For infection | 44.3 (86/194^#^)  5.2 (10/194^#^)  10.8 (21/194^#^) | 46.3 (1480/3196^#^)  4.9 (156/3187^#^)  15.3 (488/3190^#^) | 0.592  0.871  0.091 |
| Composite death/graft failure | 10.2 (28) | 8.5 (336) | 0.327 |

**Supplement 1.** **Comparison between currently and recently SARS-CoV-2 positive and negative donors in UNOS.** Values are provided as median (IQR) or % (n). ^#^Data availability as indicated. *Defined as a female donor to male recipient constellation. **Defined as “cigarette use > 20 pack years ever” in UNOS. ***Defined as acute rejection (cellular or humoral) requiring treatment. *BMI* body mass index, *CVA* cerebrovascular accident, *ICH* intracranial hemorrhage (any), *NOS* not otherwise specified, *CPR* cardiopulmonary resuscitation, *LVEF* left ventricular ejection fraction, *ICM* ischemic cardiomyopathy, *NICM* non-ischemic cardiomyopathy, *VAD* ventricular assist device, *temporary MCS* temporary mechanical circulatory support (e.g., intra-aortic balloon pump, Impella® (Abiomed®, Danvers, USA), extracorporeal membrane oxygenation), *HT* heart transplantation.
